# Supplementary material for: SRC-1 controls growth cone polarity and protrusion with the UNC-6/Netrin receptor UNC-5 in Caenorhabditis elegans
Source: PLoS One. 2024 May 21;19(5):e0295701. doi: 10.1371/journal.pone.0295701 (PMC11108135; doi:10.1371/journal.pone.0295701)
Supplement: S1 File — (DOCX) [file pone.0295701.s001.docx]

*src-1(cj293):*

*cj293* spanning read 1

TAAGGTCTCGGTAAGGAAAACTTGTAGAATTTTTCTAAATTTTATCATATTAAAATGTTGCTTAATAGTTACTC…TTTAGGTCTAAAAAACCAAAGTCTCAAATTTCGGTACTCCACCTTTTATTATCGATTTTCTTCAAATTTCAGAGTTT

*cj293* spanning read 2

GGCGAATAAGTTTTAAGGTCTCGGTAAGGAAAACTTGTAGAATTTTTCTAAATTTTATCATATTAAAATGTTGCTTAATAGTTACTC…TTTAGGTCTAAAAAACCAAAGTCTCAAATTTCGGTACTCCAGATCGGAAGAGCACACGTCTGAA

*src-1(lq185)*:

sgRNA1: 5’ GTTGCCTGTTTTCAAAAGAG 3’

sgRNA2: 5’ CCAAGTGCCTAAAAAATTGT 3’

*lq185* ssODN repair template (sequence of the *lq185* deletion):

TTTCAGAATAAATAATGGGTTGCCTGTTTTCAAAA…AGTGCCTAAAAAATTGTGCTCTTTTTTTCCAAATT

*src-1(syb7248):*

wild-type sequence:

TATTTGGAGGAGAGGAAGCTTGTGCACAGA**GATTTGGCC**GCT**CGT**AACGTTCTTGTCGGAGAT

*src-1(syb7248)* sequence:

TATTTGGAGGAGAGGAAGCTTGTGCACAGA**GCTCTTGCA**GCT**CGA**AACGTTCTTGTCGGAGAT

D381A mutation

recoded without changing coding potential
