# Supplementary material for: SRC-1 controls growth cone polarity and protrusion with the UNC-6/Netrin receptor UNC-5 in Caenorhabditis elegans
Source: PLoS One. 2024 May 21;19(5):e0295701. doi: 10.1371/journal.pone.0295701 (PMC11108135; doi:10.1371/journal.pone.0295701)
Supplement: S3 File — (DOCX) [file pone.0295701.s003.docx]

AAGCTTGCATGCaaaaacacccactttttgatctcaaatttgcacttattaagcactaaaaaccaataatttcttccctttaaaatctaaaaaaaatcttccaataacccccccactgataacaacaacaaggcatttttcactcgacgaccggtggcggagcgcacgtcttcttattcttcttcttcttcttcttttcctaccttttctatcccactttttccccggaattttgagatttgacatgatttttcagagaatttcgagttttgagaaaaaaaaaatcaaaaaagcgattttttggtcaaaagccgaaatttaaagctagtttttttggaaatatcgattttttttttgagaatttttcgattttccaaaaaaaaaatcgaaagttgtttcccgaaaagccgaaattttttttgagatatcgaaaaatcggagaatttttcgatgtttcccagaaatactaaaattttgaattttccggaaaaaaaatcgttaaaaaattagtttcttttaaagaccgaaatttaaagttttttttttggaaatatcgattttttccgagatatctaaaattgaaagaaattttcgattttctgagaaaaatacgaaaaaatcgaaaaaaaaacccaaatttcggttttttcgtaaaaaaagtcgtaaaaatgtaatttttttcctgaaaaatcggaattttttacaaaatatcggaaaatactcaaaaaaaagctgaaaatttcgattttccggagaaaaaatctttttaaaaaaatattttttttttcagaaaataagaaaagccgaaatttataactatttctccggaaattcgaaatttttaacgaaatatcggaataatttttagatttttcaagtttgactttgcgagaaaaaaatcggaaaaacctcgattcgacgccgaaaaatgctccttttcgaaaagatttttgaaatttcagaaaatcgacatgcaagcgcgctccacggcgaaatgacaacgatgatccaccgccctcaaaaagttgggtctcgttaggtatttggcggtaaaactggtaaaactccagttttgcctccaacgagacccaattttttggggcggtggtggagcgcgcttgcacaagctgaaagcatttttctgcgactcgataatattttgaaaacctgtgtcaattctcgaaattctttttttaaaaaataatcccgagcttctctcagtcctcctctatgaggatgttccttttttttggtttttcaattttttttaaaattccaaatttctgttgtgcaattcacttccccccaagaaatcccaaaaatccccagttttccccaaaaatgttccgttttcatgtgatttttcccccatttttaaaacatttttttgacttttttttaaaatgattattattattgttttctatttcatggccggtaaattattttttttctttctttttttttgctcttttttttcaagaattttcgaattgtttgaagggctgctcatctaatcttttgtcattttgttctgatgccatcatttctgagaggacctttgaagactcgtcacgaaacgggaggggggctcaagtgagcattattattattattattgtcgcaaaaagtttaccccgggctccccctggctcccctctttgagcaagggtttaagggctcattttgatgacgaattgctcattgggattatagtcacgcccctcttttggagcaactacacaactgagccacagtaatccttgggggcggggtcagtaggaccccctccggaatagggaaaagctcagttcaccgccaaaaAAGCTTGCATGCCTGCAGATGGGTTGCCTGTTTTCAAAAGAGCGGCGATCGGGAGGCAGTGATATGGGTGTTTCCGAACGGATAGATGTCTCCAGATTCCAAACGCCCCAACAACAGACAGTATTCCACGTGAATAACGGTGGAAACGAGGGAACCATCTCTCAACTAAACGGAACATCAGACGGAATGATGGGAAATGGACGTGGTGGTGGTGGTGGAGGAGGAGCACAGGAACGTGAGACTCTGGTCGCCCTCTATCCATACGATTCACGTGCCGACGGGGATCTTAGCTTTCAAAAAGGCGATGCTATGTACCTATTGGATCATTCGAATTGTGATTGGTGGTATGTTCGACACCAACGAACCGGACAGACTGGTTATGTGCCGAGGAATTTTGTTGCCAAGCAACAGACTATTGAGAGTGAAGAATGGTACGCAGGAAAGATACCAAGAAATCGTGCCGAACGACTCGTTTTATCATCACATCTACCAAAAGGAACATTCTTGATAAGAGAACGAGAAGCCGACACAAGAGAATTCGCATTGACAATTCGAGATACCGATGATCAGAGAAATGGTGGAACAGTAAAACACTACAAAATCAAACGACTGGATCACGATCAAGGATATTTCATTACAACACGACGAACATTCCGTTCTCTTCAAGAACTTGTCAGATATTATTCCGACGTTCCAGACGGCCTATGCTGTCAACTTACATTCCCAGCACCTCGCCTAGCGCCAACTCGACCCGATTTATCACATGATACACAACAGAATTGGGAAATTCCACGCAATCAGTTGCACCTGAAACGGAAGCTTGGTGATGGTAATTTCGGAGAAGTTTGGTATGGAAAATGGCGCGGAATTGTTGAGGTGGCGATTAAGACGATGAAGCCTGGTACTATGTCACCTGAAGCTTTCTTGCAAGAAGCCCAAATAATGAAGCAATGTGATCATCCGAATCTTGTAAAACTGTATGCAGTGTGTACACGTGAAGAACCATTCTACATTATAACCGAATACATGATAAATGGCTCACTTCTTCAATATCTTCGAACCGACGGAAGCACCCTCGGAATTCAGGCGCTCGTCGATATGGCCGCACAAATTGCGAACGGAATGATGTATTTGGAGGAGAGGAAGCTTGTGCACAGAcaaTTGGCCGCTCGTAACGTTCTTGTCGGAGATAAGATTTCCGGAGTTCCAGTTGTTAAAGTTGCCGACTTTGGTTTGGCGCGAAAATTGATGGAAGAGGACATTTACGAGGCACGAACCGGCGCGAAATTCCCGATTAAATGGACGGCACCCGAGGCGGCGACTTGTGGAAATTTTACGGTGAAAAGTGATGTATGGTCGTATGGTATCTTGTTATATGAGATTATGACAAAGGGACAGGTTCCGTATCCGGGAATGCACAATCGTGAAGTCGTCGAACAAGTCGAGCTGGGCTACCGTATGCCAATGCCACGTGGCTGCCCGGAGCAAATCTACGAAGAAGTACTGCTCAAATGTTGGGATAAGACGCCCGATCGGCGGCCGACTTTCGACACTTTATATCACTTTTTCGACGATTATTTCGTGTCGACGCAGCCGAATTACGCGCCACCAAGTGCCTCTAGAGGATCCCCGGGATTGGCCAAAGGACCCAAAGgtatgtttcgaatgatactaacataacatagaacattttcagGAGGACCCTTGAGGGTACCGGTAGAAAAAATGAGTAAAGGAGAAGAACTTTTCACTGGAGTTGTCCCAATTCTTGTTGAATTAGATGGTGATGTTAATGGGCACAAATTTTCTGTCAGTGGAGAGGGTGAAGGTGATGCAACATACGGAAAACTTACCCTTAAATTTATTTGCACTACTGGAAAACTACCTGTTCCATGGgtaagtttaaacatatatatactaactaaccctgattatttaaattttcagCCAACACTTGTCACTACTTTCTgTTATGGTGTTCAATGCTTcTCgAGATACCCAGATCATATGAAACgGCATGACTTTTTCAAGAGTGCCATGCCCGAAGGTTATGTACAGGAAAGAACTATATTTTTCAAAGATGACGGGAACTACAAGACACgtaagtttaaacagttcggtactaactaaccatacatatttaaattttcagGTGCTGAAGTCAAGTTTGAAGGTGATACCCTTGTTAATAGAATCGAGTTAAAAGGTATTGATTTTAAAGAAGATGGAAACATTCTTGGACACAAATTGGAATACAACTATAACTCACACAATGTATACATCATGGCAGACAAACAAAAGAATGGAATCAAAGTTgtaagtttaaacatgattttactaactaactaatctgatttaaattttcagAACTTCAAAATTAGACACAACATTGAAGATGGAAGCGTTCAACTAGCAGACCATTATCAACAAAATACTCCAATTGGCGATGGCCCTGTCCTTTTACCAGACAACCATTACCTGTCCACACAATCTGCCCTTTCGAAAGATCCCAACGAAAAGAGAGACCACATGGTCCTTCTTGAGTTTGTAACAGCTGCTGGGATTACACATGGCATGGATGAACTATACAAATAGCATTCGTAGAATTCCAACTGAGCGCCGGTCGCTACCATTACCAACTTGTCTGGTGTCAAAAATAATAGGGGCCGCTGTCATCAGAgtaagtttaaactgagttctactaactaacgagtaatatttaaattttcagCATCTCGCGCCCGTGCCTCTGACTTCTAAGTCCAATTACTCTTCAACATCCCTACATGCTCTTTCTCCCTGTGCTCCCACCCCCTATTTTTGTTATTATCAAAAAAACTTCTTCTTAATTTCTTTGTTTTTTAGCTTCTTTTAAGTCACCTCTAACAATGAAATTGTGTAGATTCAAAAATAGAATTAATTCGTAATAAAAAGTCGAAAAAAATTGTGCTCCCTCCCCCCATTAATAATAATTCTATCCCAAAATCTACACAATGTTCTGTGTACACTTCTTATGTTTTTTTTACTTCTGATAAATTTTTTTTGAAACATCATAGAAAAAACCGCACACAAAATACCTTATCATATGTTACGTTTCAGTTTATGACCGCAATTTTTATTTCTTCGCACGTCTGGGCCTCTCATGACGTCAAATCATGCTCATCGTGAAAAAGTTTTGGAGTATTTTTGGAATTTTTCAATCAAGTGAAAGTTTATGAAATTAATTTTCCTGCTTTTGCTTTTTGGGGGTTTCCCCTATTGTTTGTCAAGAGTTTCGAGGACGGCGTTTTTCTTGCTAAAATCACAAGTATTGATGAGCACGATGCAAGAAAGATCGGAAGAAGGTTTGGGTTTGAGGCTCAGTGGAAGGTGAGTAGAAGTTGATAATTTGAAAGTGGAGTAGTGTCTATGGGGTTTTTGCCTTAAATGACAGAATACATTCCCAATATACCAAACATAACTGTTTCCTACTAGTCGGCCGTACGGGCCCTTTCGTCTCGCGCGTTTCGGTGATGACGGTGAAAACCTCTGACACATGCAGCTCCCGGAGACGGTCACAGCTTGTCTGTAAGCGGATGCCGGGAGCAGACAAGCCCGTCAGGGCGCGTCAGCGGGTGTTGGCGGGTGTCGGGGCTGGCTTAACTATGCGGCATCAGAGCAGATTGTACTGAGAGTGCACCATATGCGGTGTGAAATACCGCACAGATGCGTAAGGAGAAAATACCGCATCAGGCGGCCTTAAGGGCCTCGTGATACGCCTATTTTTATAGGTTAATGTCATGATAATAATGGTTTCTTAGACGTCAGGTGGCACTTTTCGGGGAAATGTGCGCGGAACCCCTATTTGTTTATTTTTCTAAATACATTCAAATATGTATCCGCTCATGAGACAATAACCCTGATAAATGCTTCAATAATATTGAAAAAGGAAGAGTATGAGTATTCAACATTTCCGTGTCGCCCTTATTCCCTTTTTTGCGGCATTTTGCCTTCCTGTTTTTGCTCACCCAGAAACGCTGGTGAAAGTAAAAGATGCTGAAGATCAGTTGGGTGCACGAGTGGGTTACATCGAACTGGATCTCAACAGCGGTAAGATCCTTGAGAGTTTTCGCCCCGAAGAACGTTTTCCAATGATGAGCACTTTTAAAGTTCTGCTATGTGGCGCGGTATTATCCCGTATTGACGCCGGGCAAGAGCAACTCGGTCGCCGCATACACTATTCTCAGAATGACTTGGTTGAGTACTCACCAGTCACAGAAAAGCATCTTACGGATGGCATGACAGTAAGAGAATTATGCAGTGCTGCCATAACCATGAGTGATAACACTGCGGCCAACTTACTTCTGACAACGATCGGAGGACCGAAGGAGCTAACCGCTTTTTTGCACAACATGGGGGATCATGTAACTCGCCTTGATCGTTGGGAACCGGAGCTGAATGAAGCCATACCAAACGACGAGCGTGACACCACGATGCCTGTAGCAATGGCAACAACGTTGCGCAAACTATTAACTGGCGAACTACTTACTCTAGCTTCCCGGCAACAATTAATAGACTGGATGGAGGCGGATAAAGTTGCAGGACCACTTCTGCGCTCGGCCCTTCCGGCTGGCTGGTTTATTGCTGATAAATCTGGAGCCGGTGAGCGTGGGTCTCGCGGTATCATTGCAGCACTGGGGCCAGATGGTAAGCCCTCCCGTATCGTAGTTATCTACACGACGGGGAGTCAGGCAACTATGGATGAACGAAATAGACAGATCGCTGAGATAGGTGCCTCACTGATTAAGCATTGGTAACTGTCAGACCAAGTTTACTCATATATACTTTAGATTGATTTAAAACTTCATTTTTAATTTAAAAGGATCTAGGTGAAGATCCTTTTTGATAATCTCATGACCAAAATCCCTTAACGTGAGTTTTCGTTCCACTGAGCGTCAGACCCCGTAGAAAAGATCAAAGGATCTTCTTGAGATCCTTTTTTTCTGCGCGTAATCTGCTGCTTGCAAACAAAAAAACCACCGCTACCAGCGGTGGTTTGTTTGCCGGATCAAGAGCTACCAACTCTTTTTCCGAAGGTAACTGGCTTCAGCAGAGCGCAGATACCAAATACTGTCCTTCTAGTGTAGCCGTAGTTAGGCCACCACTTCAAGAACTCTGTAGCACCGCCTACATACCTCGCTCTGCTAATCCTGTTACCAGTGGCTGCTGCCAGTGGCGATAAGTCGTGTCTTACCGGGTTGGACTCAAGACGATAGTTACCGGATAAGGCGCAGCGGTCGGGCTGAACGGGGGGTTCGTGCACACAGCCCAGCTTGGAGCGAACGACCTACACCGAACTGAGATACCTACAGCGTGAGCATTGAGAAAGCGCCACGCTTCCCGAAGGGAGAAAGGCGGACAGGTATCCGGTAAGCGGCAGGGTCGGAACAGGAGAGCGCACGAGGGAGCTTCCAGGGGGAAACGCCTGGTATCTTTATAGTCCTGTCGGGTTTCGCCACCTCTGACTTGAGCGTCGATTTTTGTGATGCTCGTCAGGGGGGCGGAGCCTATGGAAAAACGCCAGCAACGCGGCCTTTTTACGGTTCCTGGCCTTTTGCTGGCCTTTTGCTCACATGTTCTTTCCTGCGTTATCCCCTGATTCTGTGGATAACCGTATTACCGCCTTTGAGTGAGCTGATACCGCTCGCCGCAGCCGAACGACCGAGCGCAGCGAGTCAGTGAGCGAGGAAGCGGAAGAGCGCCCAATACGCAAACCGCCTCTCCCCGCGCGTTGGCCGATTCATTAATGCAGCTGGCACGACAGGTTTCCCGACTGGAAAGCGGGCAGTGAGCGCAACGCAATTAATGTGAGTTAGCTCACTCATTAGGCACCCCAGGCTTTACACTTTATGCTTCCGGCTCGTATGTTGTGTGGAATTGTGAGCGGATAACAATTTCACACAGGAAACAGCTATGACCATGATTACGCCAAGCTgtaagtttaaacatgatcttactaactaactattctcatttaaattttcagAGCTTAAAAATGGCTGAAATCACTCACAACGATGGATACGCTAACAACTTGGAAATGAAAT
