# Supplementary material for: SRC-1 controls growth cone polarity and protrusion with the UNC-6/Netrin receptor UNC-5 in Caenorhabditis elegans
Source: PLoS One. 2024 May 21;19(5):e0295701. doi: 10.1371/journal.pone.0295701 (PMC11108135; doi:10.1371/journal.pone.0295701)
Supplement: Attachment — Submitted filename: Response to reviews.docx [file pone.0295701.s004.docx]

Changes made to the manuscript text are highlighted in blue.

Reviewer #1: The manuscript by Mahadik is a solid genetic study to examine the functions and genetic interactions of the UNC-5/Netrin-receptor and the SRC-1/Src kinase, which control axon guidance and growth cone properties in C. elegans. Previous work by several groups had shown that UNC-5 and SCR-1 both function in axon guidance and growth cone biology, that they physically interact, and that UNC-5 is a target for phosphorylation and regulation by SCR-1 in response to netrin signaling. One central finding of the current study is that a previously annotated null allele of SRC-1 (cj293) is in fact an in-frame deletion that does not behave as a null and may have dominant/GOF qualities. The authors generated a true null of src-1 and show that it behaves as expected based on its phenotype and genetic interactions with unc-5 mutants. They also show that a kinase dead-version of SRC-1 behaves as a dominant negative and that SRC-1 acts in the affected VD neurons (as expected).
Also included are genetic interaction data, some of which suggest refinements regarding the functions of SRC-1–UNC-5 in specific aspects of growth cone regulation.

Any immediate experimental concerns regarding this study were minor. The real issue is that the paper doesn’t provide a great deal of new information about the genes under study or advance the understanding of mechanisms controlling axon guidance in a substantial way. I do give the authors credit for taking the time to correct a mistaken description of an allele and for carrying out a very thorough set of genetic experiments. And while I generally don’t like to make judgements about impact or novelty, it’s hard to see how this study adds sufficiently to the current knowledge, either at the level of mechanistic insights or newly uncovered biology, to constitute a paper in PLoS Genetics (based on my understanding of the journal). As such, the presented work seems perhaps more suitable to journals such as G3 or PLoS One.

While this comment about impact is not relevant to publication in *PLoS One*, we disagree that this work does not advance understanding or mechanism of *src-1*. This is the first analysis of the role of SRC-1 in growth cone morphology during outgrowth, and it’s relation to UNC-5 in this process. This is a major advance and insight that gets at the cellular mechanism of SRC-1 in axon guidance (regulating growth cone polarity and protrusion), something that until this work was completely unexplored. It is disappointing that the reviewers would not see this advance and mechanism, but we accept the decision.

Minor Specific Comments:
1) The first sentence in the abstract was rather confusing and could be re-worked. In general, the abstract seemed as though it could be improved somewhat.

The introduction to the abstract has been revised as indicated in the manuscript in blue.
2) Line 144. Fig 1C seems to be the relevant panel.

Done.
3) Figure 2, 5 and related text. In general, it was difficult to clearly interpret the results involving the tissue-specific transgene rescue of src-1 given that the transgene (Punc-25::src-1) itself caused a substantial phenotype in the wild-type background.

We have modified the text to reflect that the axon guidance defects are weaker than *src-1(lq185)*. Furthermore, the growth cone defects are the opposite of the *src-1(lq185)* mutant and are consistent with overactivity of *src-1* in the growth cone.
4) Figure 3, the label in panel L was missing.

Done.
5) It was hard to make clear sense of some of the genetic interactions, such as in Figure 6A with myr-5::unc-5 and lq185.

The one result that is not consistent is that heterozygous *lq185/+* reduced growth cone area of *unc-5(ev480)*, as we noted in the text. Otherwise, the results are consistent with roles in the UNC-5 pathway and with parallel roles in other pathways, as noted in the text.

The *myr::unc-5* result, that *src-1(lq185)* does not suppress it, suggests that SRC-1 activity if not required for the function of MYR::UNC-5. The most parsimonious explanation, as stated in the text, is that the role of SRC-1 is to cause UNC-5 receptor dimerization, and this process is not required for MYR::UNC-5 dimerization.
6) In Figure 8, might want to insert the word “ventral” before filopodial protrusion in the lower left.

It is unclear where in Figure 8 this should be written.
7) Line 196, change pf to of.

Done.
8) Line 200, should Figure 6 be referenced?

Yes. Done.

Reviewer #2: Previous work has found that SRC-1 phosphorylates UNC-5 (Tong et. al, 2001) and that UNC-5 and SRC-1 function together to guide distal tip cell migration (Lee et. al, 2005). Moreover, previous work has also shown that SRC-1 is required for axon guidance (Itoh et. al, 2005). The current study suggests that SRC-1 functions with UNC-5 to inhibit growth cone size and promote axon guidance. Overall, this is a well-done set of experiments that provide more detail on the role of SRC-1 and UNC-5 in axon guidance. However, since the UNC-5/SRC-1 interaction was already known in distal tip cells and the role of SRC-1 was already known in axon guidance, this advance may be considered incremental.

Again, the advance is the mechanistic understanding of the cell biological effect of SRC-1 in the growth cone during axon guidance.

The authors characterize two alleles of src-1 and find that the src-1(cj293) and the src-1(lq185) both cause axon guidance defects and also a defect in filopodial polarity in the growth cone. However, these alleles have opposite effects on the size of the growth cone, with src-1(cj293) causing a smaller growth cone and src-1(lq185) causing a larger growth cone. The effect of src-1(lq185) on growth cone size is expected based on the known interaction between UNC-5 and SRC-1. On the other hand, the effect of the src-1(cj293) is surprising because it is the opposite of the unc-5 loss of function phenotype. The authors suggest that this may indicate that src-1(cj293) is a gain-of-function allele. These observations suggest that axon guidance and growth cone polarity can be disrupted by either src-1 loss-of function or src-1 gain-of-function.

The authors also present a series of genetic interactions to demonstrate the interaction of UNC-5 and SRC-1 in axon guidance. They find that the src-1 null allele is a dominant enhancer of the unc-5 hypomorphic allele. Since the unc-5 allele is hypomorphic, it is not clear whether this interaction would indicate same pathway or parallel pathway. They also find that the null allele of src-1 also enhances the null allele of unc-5. However, in this case the enhancement is from 95% to 100%. It is not clear whether this enhancement is robust enough to indicate a parallel pathway. For the case of growth cone size, the src-1;unc-5 double mutant is consistent with unc-5 and src-1 working in the same pathway for the control of growth cone protrusion.

Agreed that the enhancement is reaching a ceiling effect, but it is still real, suggesting parallel function in some aspect of axon guidance not assayed here.

Reviewer #3: This paper builds on earlier findings that the conserved tyrosine kinase, SRC-1, phosphorylates the Netrin  receptor UNC-5 to regulate axon guidance. In particular, this work demonstrates that axon guidance of VD/DD neurons and VD neuron growth cone polarization depend on SRC-1 function with UNC-5. Of particular importance is the documentation of the true null phenotype for src-1 based on CRISPR-generated deletion. Although these additional findings are new and also substantiate an earlier report be Lee et al.,  2005, it is disappointing that the authors do not build on their new findings to explore the mechanism of SRC-1 interaction with UNC-5. For example, this paper reports the exact genomic sequence of the src-1(cj293) used in the earlier work (Lee et al, 2005), to predict an in frame deletion that could encode a mutant SRC-1 protein with altered protein domains (see Major Revision below).  Based on a phenotypic comparison to the src-1(0) allele generated in
this work, the authors speculate that the src-1(cj293) partial deletion results in a gain-of-function SRC-1 mutant protein. But, the authors do not attempt to test this idea with biochemical experiments, for example. This is a missed opportunity that could have elevated the impact of this work. Otherwise multiple minor revisions are needed to clarify results and correct typos.

Major revisions

1.      Although additional structure-function analysis of SRC-1 is not required for a meaningful paper, I do think it is necessary to confirm that the proposed in frame src-1(cj293) deletion allele actually encodes the proposed transcript (by RT-PCR and sequencing). Even better if the edogenous mutant protein were epitope-tagged and detected by immunoblotting.

Agreed that this would be a very interesting avenue of experimentation.


 Minor revisions

Lines 148-160. Confusing to discuss src-1 alleles with opposite effects in the same paragraph. Better to discuss the authentic null allele first since its phenotype leads to the reasonable proposal that the cj293 mutation results in src-1(gf).

This section was re-written as suggested.
It would be helpful to use src-1(0) and unc-5(0)) symbols for null alleles to help readers follow the narrative

While this is a valid suggestion, we prefer to keep the nomenclature as it is.
Line 70 “To further explore define the…”

Removed “define”.
Line 71-72 What is “they” in this sentence…”If they acted in the same signaling…”

UNC-5 and SRC-1. Now indicated.
Lines 74-81…Confusing narrative about “existing src-1(cj293) allele…” This information belongs in the results section AFTER analysis of the authentic src-1 deletion allele generated in this paper.

This portion of the Introduction was re-ordered to reflect this comment.
Line 135 what is “VD/DD axon guidance”? Why not explain that DD commissures grow out earlier in the embryo and that DD and VD commissures are labeled with the unc-25::GFP marker used to assess axon guidance defects.

A sentence was added to make this more clear.
Line 157 should be “might result in…”

Done.
Line 171 should be “Transgenic…”

Done.
Lines 179-216: In the section “src-1 interacts genetically with unc-5 in axon guidance,” genetic experiments appear to test the hypothesis that src-1 and unc-5 function in a common genetic pathway for axon guidance (of VD/DD commissures) but this hypothesis is not stated. Indeed, almost all of the results here are entirely consistent with this hypothesis except for a very slight (~5%) enhancement of unc-5(0) by the src-1(0) allele. The authors appear reticent to state the obvious interpretation that these genetic results are consistent with the hypothesis that SRC-1 functions in a common pathway with UNC-5 for axon guidance. And, owing to the slight enhancement of the unc-5(0) axon guidance phenotype, src-1 may also act in an additional pathway. Otherwise, the significance of these experiments is unclear from the muddled treatment. Why are axon guidance defects presented in the same paragraphs with growth cone protrusion defects? How are these related? Please explain the
rationale for genetic interaction experiments with the unc-5(ev480) hypomorphic allele.

The hypomorphic allele was used to show that it could be enhanced by *src-1*. Enhancement of a hypomorph is consistent with action in the same pathway. This section has been significantly rearranged to reflect the reviewers’ comments and to highlight that the bulk of interactions suggests activity in a common genetic pathway.

Line 184 “unc-5(e791); src-1(lq185) double mutants displayed significantly enhanced..” relative to unc-5 or src-1 single mutants? What is conclusion from enhancement? If enhancement is from 95% -100% (i.e., only 5%) is this an important finding?

It is slight but significant, as we say in the text. This section has been revised as described above.
Line 197-198..Please explain interpretation of src-1(lq185)/+ enhancement of unc-5 alleles

Dominant enhancement is consistent with activity in the same genetic pathway. Now in the revised text.
Line 198        Figure 3B should be Figure 2B

Done.
Line 200…if increased filopodial length is not significant then its not an effect. In this case, it is actually more accurate to state that “unc-5(ev480); src-1(lq185) VD growth cones resembled those of single mutants; an apparent increase in filopodial length is not significant.”

This was removed.
Line 200-202. Why are results with src-1(0)/+ on growth cone area “not consistent with others presented here”? (Figure 6A?). “not consistent” with which specific other results? This result is unexpected given that src-1(0) does not enhance the unc-5(ev480) hypomorphic allele…but I would present this as a surprising finding at the end of this section that could be indicative of a complex role for src-1 in the axonal guidance mechanism (for example, src-1 kinase activity could have both positive and negative effects by acting on more than one target.)

Agreed. This was added.
Line218-220. “Transgenic expression (in VD/DD neurons?) of src-1(+ rescued src-1(lq185) mutants and inhibited growth cone protrusion.” Please explain the significance of these results…that src-1 is likely cell autonomous and that it acts to antagonize growth cone protrusion.

This was revised to reflect these comments.
Line 220. “determine of…” should be “determine if…”

Done.
Line 224….”unc-5(lq185)” should be “src-1(lq185)”

Done.
Line 236-238…It is not correct to state that “src-1(syb7248)/+ displayed weak axon guidance defects” because these effects are not significant.

Restated to reflect this.
Line 256. Should be “the UNC-5 cytoplasmid domain…”

Done.
Line 280. “src-1 loss of function resulting” should be “src-1 loss-of-function results…”

Done.
Line 313. “src-1(syb7248) larvae displayed strong DD axon pathfinding defects…” Where are these results? What are DD neurons?

Changed to “VD growth cones could not be identified in *src-1(syb7248)* arrested larvae.”
Discussion: The authors do not discuss the obvious possibility that the adult sterile phenotype of src-1(0) is likely indicative of a maternal effect such that the axon guidance defects presented here may be less severe than the src-1 null phenotype due to residual maternal SRC-1.

This was added to the Discussion.
