# Supplementary material for: SRC-1 controls growth cone polarity and protrusion with the UNC-6/Netrin receptor UNC-5 in Caenorhabditis elegans
Source: PLoS One. 2024 May 21;19(5):e0295701. doi: 10.1371/journal.pone.0295701 (PMC11108135; doi:10.1371/journal.pone.0295701)
Supplement: Attachment — Submitted filename: Response to reviews.docx [file pone.0295701.s005.docx]

Review Comments to the Author

Reviewer #1: I was not an original reviewer of this article. However, from my reading and assessment, Authors have addressed reviewer concerns, except confirming the expression of the older src mutant. Based on the genomic sequencing and difference in phenotyping, I am ok with this.

Reviewer #2: The Src family kinase SRC-1 has been previously demonstrated to interact physically with UNC-5 and to phosphorylate UNC-5, to guide axons. Mahadik et al. now show that SRC-1 controls VD growth cone polarity and protrusion with UNC-5 in nematodes. They generated a precise deletion of src-1 and showed that these mutants have unpolarized growth cones displaying an increase in size, a phenotype similar to unc-5 mutants. Through src-1 rescue, they show that this effect is cell autonomous. They also generated a kinase dead src-1 allele (D831A), which likely has dominant negative activity. Their genetic interaction experiments using src- 1 and unc-5 suggest that they mostly act in the same pathway on growth cone polarity and protrusion. The phenotype of an activated unc-5 (myr::unc-5) in absence of src-1 suggests that src-1 might act upstream of UNC-5 and modulate UNC-5 dimerization.

This work is novel in the analysis of SRC-1 in growth cone morphology and the connection with UNC-5 in this process. The work is well done and well quantified. However, I have one major comment that should be addressed:

The authors sequenced a previously generated SRC-1 allele harboring a deletion and they state, “if the fusion intron consisting of the 5’ splice site of exon two and 3’ splice site of exon four was removed from transcripts, and exons two and five were spliced together, the src-1 open reading frame would be retained.” From this, they further predict that a mutant SRC-1 protein would be produced. As this is purely speculative but important for their interpretation of the function of this allele (cj293) in growth cones, they need to show that it exists at the mRNA level by RT-PCR and sequencing. This is key for their interpretation as absence of mRNA production would significantly affect the interpretation of the src-1(cj293) allele. Of note, I am not asking here for biochemical experiments, I am simply asking to check that the mRNA that the authors predict is indeed generated. This is a straightforward experiment that shouldn’t take very long to perform.

This is a good point the reviewer makes. We tried several times to amplify a cDNA from *src-1(cj293)* spanning the predicted exon 2 to exon 5 splice and were unsuccessful. Possibly this splice does not occur. On Wormbase, a *src-1D* isoform is described consisting of only the last two exons encoding the kinase domain. *cj293* does not affect the coding region of predicted *src-1D* isoform. Possibly, production of *src-1D* without the other isoforms in *cj293* results in the gain-of-function phenotype. We now mention this as an alternate possibility in the manuscript.
